# Epidemiology of in-hospital trauma deaths in a Brazilian university hospital

**DOI:** 10.1186/1471-227X-14-22

**Published:** 2014-10-31

**Authors:** Adriano D Trajano, Bruno M Pereira, Gustavo P Fraga

**Affiliations:** 1 Division of Trauma Surgery, Department of Surgery, School of Medical Sciences, University of Campinas, Campinas, SP, Brazil

**Keywords:** Injuries, Trauma, Mortality, Violence, External causes

## Abstract

**Background:**

The analysis of patterns of trauma deaths may improve the evaluation of a trauma system and identify areas that may benefit from more resources. The objective of this study was to analyze the epidemiology of trauma deaths in a Brazilian university hospital in order to assess the profile of these fatalities over a 16-year period.

**Method:**

Retrospective study of time series using database records. The research subjects were in-hospital deaths from external causes during the years 1995, 2000, 2005 and 2010. The following variables were analyzed: cause of injury, trauma scores, time and cause of death.

**Results:**

467 cases were studied, being 325 patients (69.6%) admitted with signs of life and 142 (30.4%) considered dead on arrival. The mean age was 35.35 ± 18.03 years. 85.4% were males. Blunt trauma occurred in 73.0% of cases and penetrating mechanism in 27.0%. There was a significant increase (p < 0.001) in deaths from motorcycle crashes over the years, which went from 7.3% in 1995 to 31.5% in 2010. In contrast, there was a significant decrease (p = 0.030) in firearm-injury victims; from 21.0% in 1995 to 9.6% in 2010. About 60% of deaths occurred less than 24 hours after admission. The main causes of death were lesions of the central nervous system (56.3% of the total), followed by hemorrhagic shock (18.1%) and sepsis/multiple organ dysfunction syndrome (17.1%). The mean Injury Severity Score (ISS) of patients with signs of life was 26.41 ± 9.00, 71.3% of whom had ISS >25. The mean Revised Trauma Score (RTS) was 5.24 ± 2.05. Only 25.8% of the deaths had TRISS <0.50.

**Conclusion:**

There was a shift in the profile of causes of death from trauma in this university teaching hospital, with a large decrease in penetrating injuries and a higher incidence of deaths of motorcycle riders.

## Background

Globally, external causes of death are a major challenge to public health authorities [1]. They account for more than five million deaths annually worldwide and cause temporary or permanent disability to millions more, representing about 9% of global mortality. For every death, it is estimated that there are dozens of hospitalizations, hundreds of visits to emergency and thousands of medical appointments [2]. External causes, including trauma, mainly affect people from 5 to 44 years old, usually males, living in poor and developing countries [3].

In 2010, Brazil registered 143,256 deaths from external causes, 21% more than in 2000 (118,397 deaths). External causes account for 12.9% of registered deaths in the country, being the third cause of death among Brazilians, losing only to cardiovascular diseases (29%) and cancers (16%) [4]. The pattern in Brazil differs from elsewhere in the world because most deaths are caused by homicide or are related to traffic accidents, unlike most WHO-member countries, where 51% of deaths related to external causes are suicides and 11% are due to warfare [5].

Studies have shown that the analysis of the epidemiology of traumatic deaths can improve the assessment of a trauma system and identify the critical areas that may benefit from education provision, research and allocation of resources. It is known that the distribution of times and locations of in-hospital deaths from trauma are influenced by cause of injury, age and injured body areas [6-12].

Lesions of the central nervous system (CNS) appear as the main cause of death among trauma patients, being present in about 50% of the victims, and with a mortality peak in 24 to 72 hours after hospital admission. Hemorrhagic shock is the second most frequent cause of death, ranging from 20% to 40% in some series, depending on the rate of penetrating trauma victims at the specific region [6,7,10-12]. Traumatic deaths occurring beyond the expected time have been used as a parameter to assess the quality of care of trauma patients. Exsanguination is the leading cause of in-hospital deaths and is deemed as preventable by the delay between diagnosis and definitive treatment. Several studies confirm that the percentage of avoidable deaths from trauma is significantly higher when there is no adequate structure for treatment and when there is no medical expertise available to work on issues related to trauma patients [13-16].

In Brazil there is only rudimentary information on mortality due to lack of programs to collect data and assess information related to trauma. Trauma records are essential to guide the decisions and actions relevant to trauma victims and to serve as a link between what needs to be known about the causes and outcomes of trauma, and the development of local, regional and national intervention plans. A computerized system is essential to store these data, so that information can be easily accessed and the results analyzed practically and rapidly. Knowledge of this information is crucial for the mapping of trauma incidence in the country [17-20].

The aim of this study is to analyze time-series of deaths by trauma at a University Teaching Hospital (HC-UNICAMP), according to the causes of injury, time and causes of in-hospital deaths, injury severity and body-affected areas. We hypothesized that the pattern of the trauma deaths has changed in recent years at our specific hospital.

## Methods

The HC-Unicamp has about 500 beds and serves patients predominantly with high complexity health issues from within the entire metropolitan area of Campinas, which corresponds to a population of about 3 million inhabitants. It is one of the referenced centers for trauma patients in this region. Currently the city of Campinas has 1.2 million inhabitants, and has two other public hospitals (with 200 beds each) for trauma patients. The Division of Trauma (DCT) of HC-UNICAMP was established in 1987 and is responsible for traumatic and non-traumatic surgical emergencies for adults, admitting about 5,000 trauma patients per year. We retrospectively reviewed the charts of all in-hospital deaths from external causes (codes V01-Y98 in Chapter XX of the 10th revision of International Classification of Diseases, ICD-10) during the years 1995, 2000, 2005 and 2010. The 5-year intervals enabled the demonstration of death trends. We included all patients admitted to the emergency room of HC-Unicamp who died, including those dead on arrival. All lesions present, major or minor, were considered. In-hospital deaths by drowning, poisoning, burns, falling with isolated long-bone fracture and medical complications were excluded. Patients with incomplete charts, mostly from the year 1995, were also excluded. The causes of death were determined by clinical evidence, laboratory tests, imaging methods and surgical findings. In Brazil, by law, medico-legal autopsies are performed in all cases of sudden, suspicious or external-cause-related deaths, although there were no autopsy data analyses in this study.

Patients declared dead at the scene while under pre-hospital care were not transported to the hospital. Some patients had signs of life (SOL) in the ambulance or helicopter and were admitted to the trauma bay with traumatic cardiopulmonary arrest (no pulses, no electrical activity) and subsequently considered dead on arrival (DOA). These patients, except for those with stab wounds in the cardiac area, were not submitted to Emergency Department thoracotomy and were declared deceased.

The pre-hospital care system in the metropolitan area of Campinas is mature, and few patients are transported using their own means. Data regarding the pre-hospital care of all patients were obtained from all years since the year 2000.

For each patient admitted with SOL, a data register was created, containing the following information: age, gender, transport mode to hospital, pre-hospital time, time between admission and death (death less than 24 hours was called early death), length of stay, location of death inside the hospital, cause of injury, cause of death, surgical procedures performed, systolic blood pressure (SBP), respiratory rate (RR) and Glasgow Coma Scale (GCS) on admission. Severity of injury was determined using the trauma scores: Revised Trauma Score (RTS), Abbreviated Injury Scale (AIS), Injury Severity Score (ISS) and the probability of survival (TRISS).

Causes of death were classified as lesions to CNS, hemorrhagic shock (HS), sepsis and multiple organ dysfunction syndrome (MODS). The locations of death considered were DOA, emergency room, operative room, intensive care unit (ICU) and ward (in some cases there were not ICU beds available for all trauma patients).

The research protocol of this study was approved without restrictions by the Committee of Research Ethics and Institutional Review Board (IRB), School of Medical Sciences, Unicamp, protocol number 652/2011. The authors had complete access to patients’ charts and kept the database information anonymous.

Statistical analyses were performed using chi-square test, Fisher exact test, Mann–Whitney test and the Cochran-Armitage trend test. Analyses of univariate and multivariate logistic regressions were used to identify the risk factors of death within 24 hours of admission. P values of less than 0.05 were considered significant. All data were analyzed using the SAS system (Release 8.2, SAS Institute Inc).

## Results

From a total of 7,258 deaths registered in HC-Unicamp as a result of various causes and pathologies in the defined periods (years 1995, 2000, 2005 and 2010), 549 deaths (7.56% of the total) were caused by trauma. During these years, there was a relative and absolute decrease in the cases of traumatic deaths, falling from 240 deaths (9.2% of the total) in 1995 to 89 deaths (5.0% of the total) in 2010. Looking at the number of deaths from trauma in relation to the number of hospital admissions for trauma ICD, there was a decrease from 3.1% in 1995 to 1.4% in 2010. From a total of 549 deaths due to trauma, 82 were excluded (15.0%) due to incomplete data in patients charts.

**Figure 1:**
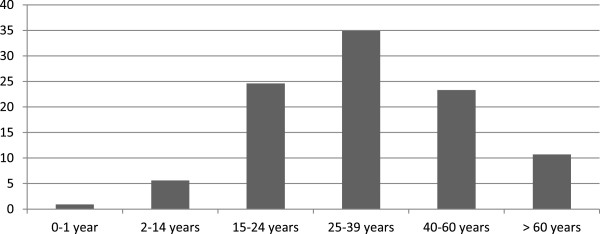
Percentage of cases according to age group.

467 cases (85.0% of the total sample) were studied, the vast majority being male (85.4%, n = 399). The average age of the patients was 35.35 ± 18.03 years, varying from 1 to 99 years of age (Table 1). It was observed that the predominant age group was 25–39 years, with 163 patients (34.9%), followed by the age group 15–24 years (24.6%) (Figure 1).

**Table 1:** Study population characteristics (N = 467)

	**Frequency (N and %)**	**Average ± SD**
Age (years)		
≤ 60	417 (91.3)	35.35 ± 18.03
> 60	50 (10.7)	
Gender		
Male	399 (85.4)	
Female	68 (14.6)	
Mechanism of injury		
Blunt	341 (73.0)	
- Running over	113 (24.2)	
- Motorcycle crash	70 (15.0)	
- Car crash	66 (14.2)	
- Fall	58 (12.4)	
- Bicycle	17 (3.6)	
- Assault	8 (1.7)	
- Others	9 (1.9)	
Gunshot wound	108 (23.2)	
Stab wound	18 (3.5)	
Signs of life on arrival		
Yes	325 (69.6)	
No (DOA)	142 (30.4)	
Revised Trauma Score (RTS)		5.24 ± 2.05
Injury Severity Score (ISS)		26.41 ± 9.00

**Figure 2:**
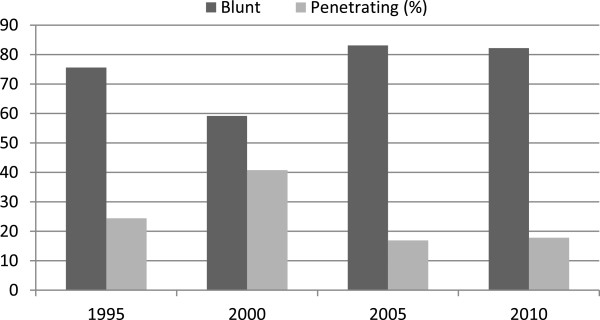
Types of trauma, in percentage, over the years.

The most common type of injury was blunt trauma with 341 cases (73.0%) (Table 1), but there was no statistical difference between the mechanisms of trauma over the years (p = 0.181) (Figure 2).

Of the study group, 142 (30.4%) were declared DOA and 325 cases (69.6%) were admitted with SOL to the emergency room. There is a statistical difference between the SOL and DOA groups over time, and a decrease in the DOA group (p < 0.001). Comparing variables between the SOL and DOA groups, there is no statistical difference between the groups for: age (lower in DOA, 32.31 ± 15.36 years, versus 36.68 ± 18.94 years, p = 0.031), method of pre-hospital transport (higher frequency of rescue and own means in DOA), type of trauma (higher frequency of penetrating in DOA), surgery performed (lower frequency in DOA), and cause of injury (higher frequency of motorcycles, bicycles and falls in SOL and of gunshots in DOA). Comparative analysis of the DOA and SOL groups showed that the age range ≤60 years was statistically significant (p = 0.019) in the DOA group.

Regarding the causes of trauma, in general, the most frequent were: PHBC with 113 cases (24.20%) and GSW with 108 cases (23.13%), but over the years there was a significant increase (p < 0.001) of motorcycle accidents and stab wounds (p = 0.030) in the total sample.

Of the 325 patients (69.6%) who were admitted with SOL to the emergency room, the mean ISS was 26.41 ± 9.00, with 93.1% (303 cases) having ISS greater than 15. The mean RTS was 5.24 ± 2.05. Regarding the body segments, the most affected (with AIS score greater than or equal to four) were: head with 222 cases (68.3%), followed by chest with 44 cases (13.5%) and abdomen with 39 cases (12.0%). The average TRISS in every sample year was 0.68 ± 0.33, and 74.1% (241cases) had TRISS ≥50. 29.2% of patients (95 cases) suffered multiple traumas (three or more body segments with affected AIS score), 60.3% (169) of patients were admitted with GCS less than or equal to 8, while 27.6% (90) of the patients were admitted with SBP less than 90 mmHg. The most common causes of death were: lesion of the CNS with 183 cases (56.3%), hemorrhagic shock (HS) with 59 cases (18.1%) and sepsis and MODS with 56 cases (17.1%) together. Patients who died of HS had the following body segments most affected: abdomen (32.8%), chest (28.4%) and pelvis (14.9%).

With respect to the time of death after hospital admission, considering the dead upon arrival, we found that 267 patients (57.18%) died within 24 hours of hospital admission. We performed a comparative analysis between the patients who died within 24 hours (early death) and those who didn’t. Statistically significant (p < 0.001) were the early deaths in penetrating injuries, SBP less than 90 mmHg on admission, chest and abdomen injury with AIS ≥ 4, hemorrhagic shock as cause of death and TRISS < 0.50. The CNS injury mortality peaked within 24–72 hours of hospital admission (33.9%) and the peak of death due to HS occurred between 6–24 hours (37.3%) and sepsis/MODS after 7 days (89.3%).

Tables 2 and 3 present the results of analyses of univariate and multivariate logistic regression to study the risk factors associated with early death in the SOL group. From the results of the multivariate analysis with Stepwise criterion variable selection, it appears that the variables place of death, head injury, cause of death and avoidable death (TRISS) were associated significantly with early death in the SOL group. The patients at greatest risk of death within 24 hours were: those in the emergency room and operating room (risks of 4.6 and 14.2 times higher, respectively), those with head injury with AIS < 4 (7.1 times greater risk), those whose trauma causes were CNS injury/HS and acute respiratory failure (risks of 30.4 and 17.7 times, respectively), and those presenting avoidable death (TRISS <0.50) (risk of 3.3 times higher).

**Table 2:** Significant risk factors for early death (SOL) using univariate logistic regression analysis

Variable*	**Categories**	**P-value**	OR**	**IC 95% OR**
Mechanism of Trauma	Blunt (ref.)	—	1.00	—
	Penetrating	<0.001	3.20	1.81 – 5.67
Cause of death	Sepsis/MODS (ref.)	—	1.00	—
	CNS/Shock	<0.001	22.83	5.45 – 95.69
	Respiratory failure	0.001	27.00	3.74 – 195.17
	Others	0.003	18.00	2.71 – 119.79
Place of death	Ward (ref.)	—	1.00	—
	Emergency room	<0.001	5.96	3.18 – 11.18
	Operating room	<0.001	150.04	8.91 – 2527.03
	ICU	0.123	0.59	0.31 – 1.15
PAS	> 89 (ref.)	—	1.00	—
	≤ 89	<0.001	4.09	2.46 – 6.81
Head Injury	< 4 (ref.)	—	1.00	—
	≥4	<0.001	0.28	0.17 – 0.46
Chest Injury	< 4 (ref.)	—	1.00	—
	≥ 4	<0.001	4.20	2.12 – 8.29
Abdomen Injury	< 4 (ref.)	—	1.00	—
	≥ 4	0.006	2.59	1.31 – 5.13
Avoidable death TRISS	≥ 0,50 (ref.)	—	1.00	—
	< 0,5	<0.001	4.01	2.38 – 6.76
RTS	Continuous variable	<0.001	0.782	0.698 – 0.877
TRISS	Continuous variable	<0.001	0.155	0.076 – 0.315

*No early death (n =200); With early death (n =125). Ref: reference level. **OR = Reason for risk of early death; IC 95% OR = 95% confidence interval for OR (Odds Ratio).

**Table 3:** Multivariate logistic regression analysis for early death (SOL)

Selected variables*	**Categories**	**P-value**	OR**	**IC 95% OR**
1. Place of death	Ward (ref.)	—	1.00	—
	Emergency room	<0.001	4.64	2.25 – 9.54
	Operating room	<0.001	14.19	3.00 – 67.16
	ICU	0.168	0.59	0.28 – 1.25
2. Head Injury	< 4 (ref.)	—	1.00	—
	≥ 4	<0.001	0.14	0.07 – 0.29
3. Cause of death	Sepsis/MODS (ref.)	—	1.00	—
	CNS/Shock	<0.001	30.39	6.60 – 140.00
	Respiratory failure	0.038	17.67	1.17 – 267.44
	Others	0.101	6.58	0.70 – 62.22
4. TRISS	≥ 0,50 (ref.)	—	1.00	—
	< 0,5	<0.001	3.31	1.64 – 6.68

*No early death (n = 200); With early death (n = 125). Stepwise selection criterion variables. Ref: reference level. **OR = Reason for risk of early death; IC 95% OR = 95% confidence interval for OR (Odds Ratio).

Regarding the location of death in the hospital, most patients died in the ICU or ward. It was observed that death from penetrating trauma was statistically significant in the operating room and death from blunt trauma in the ICU (p = 0.014). The average hospital stay was 9.68 ± 48.67 days.

In the years 2000, 2005 and 2010, from a total of 222 patients (47.53% of the total sample), the hospital received 89 patients (40.09%) brought in by EMS (rescue services) and 133 patients (59.90%) were brought in by non-rescue services (ambulances, transferred from other Campinas health services). The mean pre-hospital times in the “rescue services” group were 55.38 ± 38.43 minutes in 2000, 44.40 ± 30.86 minutes in 2005 and 46.00 ± 30.98 minutes in 2010. The mean pre-hospital times in the “ambulance” group were similar within the time series with an overall mean of 145.61 ± 165.70 minutes. If we compare the “rescue services” and “ambulance” groups, there were no statistical differences between the mechanisms of injury (blunt versus penetrating), causes of death (CNS injury/HS versus sepsis/MODS), severity scores (ISS ISS ≤ 15 versus ISS > 15), head injuries (AIS < 4 versus AIS ≥ 4) and causes of trauma.

## Discussion

This study not only reveals the complex scenario of trauma in a specific local region, but that trauma remains the leading cause of death in the younger population. We observed a decrease in the cases of traumatic deaths recorded in the hospital for various diseases during the years studied. Traffic accidents and violence accounted for about 9% of in-hospital deaths in 1995, falling to 5% in 2010. This also explains the decrease in the number of trauma admissions in HC-Unicamp by around 30% during the same period.

In this study, 66% of recorded deaths occurred in patients under 40 years of age with the predominant age range being 25–39 (34.9%). The vast majority of deaths were male (85.4%) which is also observed in literature [21-23]. It can be noted that deaths caused by penetrating trauma occurred in younger individuals, but in the elderly, over 60 years of age, it was noted that almost all patients were victims of blunt trauma, mainly caused by road accidents. This also agrees with international literature [6,15].

Regarding the causes of injury, there was a significant increase in deaths of motorcycle riders over time, increasing from 7.3% in 1995 to 31.5% in 2010. Carrasco and colleagues [21] studied all fatal motorcycle crashes between 2001 and 2009 in Campinas, Brazil, and in 479 autopsies it was observed that the number of deaths from traffic accidents exceeded that of homicides and other external causes of death, and motorcycles play a significant role in these statistics. Gawryszewski and colleagues [23], studying responses to land transport accidents in the State of São Paulo, noted that motorcycle accidents were the majority, representing 29.8% of cases, followed by automotive (25.7%) and pedestrians (24.1%). Marín-León and colleagues [24], studying the trend of traffic accidents in Campinas, found an increase of 241% in the motorcycle fleet in just over a decade, and which represented nearly 50% of all fatal accidents on public roads in 2008. Reichenheim and colleagues [25] observed that the proportion of deaths from motorcycles, within the total of traffic-related deaths in Brazil, rose from 4.1% in 1996 to 28.4% in 2007, showing an increase in risk of 820%. This study concluded that the increase in motorcycle deaths is due to the immense expansion of the fleet in the country, which doubled between 2001 and 2005.

The deaths considered as dead on arrival (DOA) accounted for about 30% of the total. We observed a significant decrease (p < 0.001) in the number of cases of DOA over time, which can be mainly explained by the decrease in the number of gunshot victims in Campinas, where the homicide rate fell from 46.2 per 100,000 in 2000 to 14.5 per 100,000 in 2010 [24]. The high homicide rates were explained by the strong associations between social disorganization, due to rapid urbanization since the 1990s, arms and drugs trafficking, illegal possession of weapons and police violence. In the last decade, there has been a decrease in mortality from homicide in southeastern Brazil, mainly due to economic growth associated with disarmament policies and public security [25]. In the study on motorcycle deaths in Campinas, it was found that 50.3% of the victims died before receiving adequate medical attention [21].

As for the times of death, the peak of late mortality was observed after 72 hours when 25% of the deaths occurred due to sepsis and MODS. The in-hospital deaths, which occurred within less than 24 hours, accounted for 57% of the sample. Comparing the data with the Demetriades [15] study, there was a smaller number of deaths from 1 to 24 hours and a greater number after 72 hours. This may represent a lack of organized trauma system care in the region. The deaths that took place within 24 hours were statistically significant (p < 0.001) in patients with penetrating injury, SBP less than 90 mmHg on admission, AIS ≥4 in the chest and abdomen, HS as a cause of death and TRISS < 0.50. Patients at the greatest risk of death within 24 hours were: those in the local emergency room and operating room (risk 4.6 and 14.2 times higher, respectively), those with head injury with AIS < 4 (7.1 times greater risk), those with CNS injury/hemorrhage shock and acute respiratory failure (risks 30.4 and 17.7 times greater, respectively), and those with TRISS < 0.50 (3.3 times greater risk). As for the location of death, it is understandable that is higher in the OR because critical patients with HS are transferred to this area quickly. It was also high in the ED, compared with the ICU and ward.

Periodic assessment of time and place of traumatic deaths can provide a valuable assessment of a trauma system or trauma center [15]. The time of traumatic deaths was described in 1977, during the development of the American trauma system. Baker and colleagues [8] conducted a study of trauma deaths in the city of San Francisco during a period of one year when they analyzed 437 autopsies and described the classic trimodal distribution of deaths by trauma. The authors concluded that the deaths occurred during one of three peaks: immediately, early hospital (less than 48 hours) and late hospital (more than 48 hours). In 1992, Sauaia and colleagues [9] reevaluated the study by the Baker group and concluded that there was no longer a trimodal distribution. There was a shift from immediate to early-hospital deaths that was attributed to the improvement of pre-hospital care. Between 1993 and 2002, Demetriades [15] also showed that the trimodal distribution did not apply to his trauma system and that the improvement of pre-hospital emergency service in his region over the years increased the rate of admission of patients considered "in extremis" (extremely serious patients) arriving at the trauma center, but he also noticed the disappearance of the peak of late mortality caused by sepsis and MODS, as cited by the Baker study. This is primarily due to the improvements in pre-hospital, hospital and intensive care. The authors advocated that a more efficient emergency medical service could have been responsible for this change. It is known that cause of injury, age and injured body region are all factors having an influence on the temporal distribution of trauma deaths, and related statistics would be a reflection of improving care in each region, and of the capacities of their trauma systems. With regard to the location of death, 28.9% of patients died in the ward beds, 19.9% in the Intensive Care Unit (ICU), 15.2% in the emergency room and 5.5% in the operating room. This result does not coincide with existing literature, although the metropolitan region of Campinas is one of the most socio-economically developed in the country, and has a low percentage of deaths occurring post-24-hours in intensive care beds. The lack of beds in the ICU is due to a high demand of patients with diverse pathologies treated in a multidisciplinary hospital, chronic underfunding and a lack of specific beds for trauma patients.

With regard to the causes of death, injuries to the central nervous system resulting from traumatic brain injury were the most frequent at 56.3% of the total. Brain injuries also appear as the main cause of death in other publications with rates of between 21% and 71% [21,26,27]. In this study, death by head trauma occurred mainly between 24 and 72 hours after trauma. HS was the second cause of death (18.1%), with a peak occurring between 6 and 24 hours. The areas most affected by bleeding were abdomen (32%), chest (28%) and pelvic region (10%). As reported in literature, some studies have shown that deaths from hemorrhage occur mainly in the first 6 hours after injury and others in up to 24 hours [15,16,26]. Sepsis and MODS corresponded, respectively, to the third and fourth most frequent causes of death, totaling 17.1% and occurring 7 days after trauma. There was a significant increase (p = 0.023) of sepsis/MODS compared to CNS injury/HS over the years studied, and this caught our attention, since, in literature, there has been a decrease. This may be related to the lack of organization within the trauma care system, and consequently to a long pre-hospital time for patients transferred from other health services – the group called “ambulance”. About 70% of patients taken by pre-hospital emergency care – the “rescue services” group - died within 72 hours, which corroborates the thesis that death from sepsis/MODS was higher in the “ambulance” group. The most common cause of death in this group remains CNS injury at 30.1% (28 cases), followed by HS at 23.6% (22 cases) and sepsis/MODS at 11.8% (11 cases). This concurs with international literature [14,16,21,27]. Comparing the causes and locations of death, the OR was the most common place for patients who died of HS in 30.5% of cases, and for those who died of CNS injury or sepsis/MODS, the ward beds were the most common places of death, at 44.8% and 60.7% respectively.

With respect to rates of trauma, the neurological level on admission showed a lower RTS, and 60.3% of patients admitted with signs of life (SOL) had a GCS less than or equal to eight. In this study, the RTS analysis resulted in a mean of 5.24 ± 2.05. The analysis of ISS resulted in a mean of 26.41 ± 9.00, having 71.3% with a value greater than 25 (considered very severe cases). The mean ISS amongst the blunt trauma cases was 26 and for the penetrating cases, 27. Other publications have described an average of 38–40 in cases of deaths by trauma [28,29]. The lower average in our hospital may be explained by the fact that in most of the other publications, patients with isolated lesions were excluded, even if they were potentially fatal, and some studies took into consideration the rates of pre-hospital deaths, which are gathered by way of autopsies.

Of the 325 patients for which the trauma indices were calculated, in 135 (41.5%), injuries occurred in a single body segment, and only 91 patients (28.0%) had lesions in three or more segments. For the body area with AIS (Abbreviated Injury Score) greater than or equal to four, the head was the part most affected, with 222 cases (68.3%), followed by the chest with 44 cases (13.5%) and abdomen with 39 cases (12.0%). The TRISS results, showing an average of 0.68 ± 0.33 and with only 25.8% of patients showing TRISS < 0.50, revealed the limitation of this method in this sample, when analyzed in isolation. The initial objective of TRISS was to develop norms for the treatment of trauma that could be adopted in hospitals, in order to ensure a certain quality of care. Thus it would be then possible for hospitals to compare their results for groups of patients with similar severity, and identify, for further analysis, patients who died unexpectedly. The identification of these patients, whose results deviate from the established norm, allows assessment of preventable or possibly-preventable deaths, in an attempt to identify any eventual flaws in diagnosis, interpretation or application of techniques, motivating the medical staff for change in eventual conduct [18].

Many studies have shown that an organized system of trauma care is essential in reducing mortality and sequelae resulting from trauma [30-33]. Thus, measures ranging from prevention to the investment of resources in the health sector are necessary. Fraga [18] points out that, in Brazil, there is not yet an organized system of trauma care with coverage of the different phases of care, and also that there are no epidemiological studies or trauma registries at the municipal, state and federal levels. There is little information regarding pre-hospital care, and a disintegration among hospitals of different complexities and the legal Medical Institute, leads to a lack of information which could be used for a comprehensive study on the reasons of death from external causes. Anyway, in our region, systems of trauma care are still undergoing maturation, requiring more research, education and financial resources, in order to reduce deaths from trauma.

This study has the limitation of analyzing the deaths in only one hospital in Campinas, and it doesn’t represent the total mortality for external causes in the city. Other limitations were: it was retrospective (15% of patients were excluded due to incomplete data) and no autopsy analyses were considered.

## Conclusions

It is concluded that the deaths due to trauma occurred predominantly in young people and those of the male gender, and that penetrating trauma was more common among the young and blunt trauma the elderly, where being run over constituted the major cause of death. In this hospital there was a decrease in cases of traumatic deaths over the years, associated with the decline in the number of hospital visits for trauma and gunshot victims. There was a significant increase in deaths by motorcycle accident, which in 2010, became the leading cause of traumatic death. The deaths occurring within 24 hours were associated with penetrating injuries, SBP less than 90 mmHg on admission, AIS greater than or equal to four in the chest and abdomen, hemorrhagic shock and TRISS below 0.50. CNS injury remains the most common cause of death, and hemorrhagic shock was the second most frequent, especially of victims of penetrating trauma. A late mortality peak persists in the present study, and was caused by sepsis and MODS, which may be a reflection of a lack of organization and maturation within the trauma care system in which the HC-Unicamp finds itself.

### Key messages

• There was a decrease in cases of traumatic deaths over the years, associated with the decline in the number of hospital visits for trauma and gunshot victims.

• There was a significant increase in deaths by motorcycle accident, which in 2010 became the leading cause of traumatic death.

## Abbreviations

AIS: Abbreviated injury scale; CNS: Central nervous system; DOA: Dead on arrival; DCT: Division of trauma surgery; GCS: Glasgow coma scale; HC: Unicamp University Hospital of the University of Campinas; HS: Hemorrhagic shock; ICD: International classification of diseases; ICU: Intensive care unit; ISS: Injury severity score; MODS: Multiple organ dysfunction syndrome; RR: Respiratory rate; RTS: Revised trauma score; SOL: Admitted with signs of life; SPB: Systolic blood pressure; TRISS: Trauma score and injury severity score; WHO: World Health Organization.

## Competing interests

The authors declare that they have no competing interests.

## Authors' contributions

ADT participated in the acquisition, statistical analysis and interpretation of data, participated in the study’s design and drafted the manuscript. GPF conceived the study, and participated in its design and coordination and helped to draft the manuscript. BMP contributed to the study’s design and the revision of the manuscript. All authors read and approved the final manuscript.

## Authors' information

ADT: Division of Trauma Surgery, Department of Surgery, School of Medical Sciences, University of Campinas (Unicamp), Campinas, São Paulo, Brazil.

GPF: Professor, Head, Division of Trauma Surgery, Department of Surgery, School of Medical Sciences, University of Campinas (Unicamp), Campinas, São Paulo, Brazil; and Postdoctoral Fellow at University of California, San Diego (UCSD).

BMP: Division of Trauma Surgery, Department of Surgery, School of Medical Sciences, University of Campinas (Unicamp), Campinas, São Paulo, Brazil.

## Pre-publication history

The pre-publication history for this paper can be accessed here:

http://www.biomedcentral.com/1471-227X/14/22/prepub
